# How Different Pathologies Are Affected by IFIT Expression

**DOI:** 10.3390/v15020342

**Published:** 2023-01-25

**Authors:** Justin H. Franco, Saurabh Chattopadhyay, Zhixing K. Pan

**Affiliations:** Department of Medical Microbiology and Immunology, University of Toledo College of Medicine and Life Sciences, Toledo, OH 43614, USA

**Keywords:** IFIT protein, type-I IFNs, IFNβ, cancer, viral sepsis

## Abstract

The type-I interferon (IFN) system represents the first line of defense against viral pathogens. Recognition of the virus initiates complex signaling pathways that result in the transcriptional induction of IFNs, which are then secreted. Secreted IFNs stimulate nearby cells and result in the production of numerous proinflammatory cytokines and antiviral factors. Of particular note, IFN-induced tetratricopeptide repeat (IFIT) proteins have been thoroughly studied because of their antiviral activity against different viral pathogens. Although classically studied as an antiviral protein, IFIT expression has recently been investigated in the context of nonviral pathologies, such as cancer and sepsis. In oral squamous cell carcinoma (OSCC), IFIT1 and IFIT3 promote metastasis, while IFIT2 exhibits the opposite effect. The role of IFIT proteins during bacterial/fungal sepsis is still under investigation, with studies showing conflicting roles for IFIT2 in disease severity. In the setting of viral sepsis, IFIT proteins play a key role in clearing viral infection. As a result, many viral pathogens, such as SARS-CoV-2, employ mechanisms to inhibit the type-I IFN system and promote viral replication. In cancers that are characterized by upregulated IFIT proteins, medications that decrease IFIT expression may reduce metastasis and improve survival rates. Likewise, in cases of viral sepsis, therapeutics that increase IFIT expression may improve viral clearance and reduce the risk of septic shock. By understanding the effect of IFIT proteins in different pathologies, novel therapeutics can be developed to halt disease progression.

## 1. Introduction

### 1.1. The Interferons (IFNs)

The innate immune system serves as the initial response against infection, acting as the first line of defense before activation of the adaptive immune system. When exposed to viral pathogens, the innate immune system relies on a class of cytokines called interferons (IFNs) to initiate an antiviral defense. Typically, IFN-mediated activation of the Janus kinase (JAK)/signal transducer and activator of transcription (STAT) pathway results in the upregulation of hundreds of antiviral proteins [1,2]. There are three main classes of IFN: type-I, type-II, and type-III [1,3,4,5]. Although each class of IFN is expressed in response to a viral infection, they differ in regard to their cell of origin and action [1,3,4,5]. Type-I IFNs are the most abundant class of IFN and include two predominant cytokines: IFNα and IFNβ [1,3,4,5]. IFNα exists as multiple isoforms and is predominantly secreted by plasmacytoid dendritic cells, while IFNβ is a single protein that can be secreted by most cell types [1,3]. Unlike type-I IFNs, type-II and type-III IFNs are produced in a narrower subset of cells, namely natural killer and epithelial cells, respectively [4,5]. Type-II IFNs represent only a single cytokine, IFNγ, whereas type-III IFNs include multiple subtypes of IFNλ [4,5].

### 1.2. Pattern Recognition Receptors (PRRs)

Due to its presence in most cell types, the signaling pathway of the type-I IFN response will be summarized further in this review. Activation of the innate immune system occurs when infectious agents are detected by cellular pattern recognition receptors (PRRs) that are located in the plasma membrane, endosome, or cytoplasm [3,6,7,8]. Different categories of PRRs can interact with specific pathogen-associated molecular patterns (PAMPs), such as bacterial lipopolysaccharide (LPS), fungal β-1,3-glucan, and viral nucleic acids [3,7,8]. PRRs can be broadly categorized into five groups: retinoic acid-inducible gene-I (RIG-I)-like receptors (RLRs), Toll-like receptors (TLRs), nucleotide oligomerization domain (NOD)-like receptors (NLRs), C-type lectin receptors (CLRs), and absent in melanoma-2 (AIM2)-like receptors (ALRs). The type-I IFN response is classically triggered by PRRs that recognize viral RNA or DNA, such as RLRs and endosomal TLRs [7,8].

### 1.3. RIG-I-like Receptor (RLR) Signaling

RLRs are located in the cytoplasm of most cells and function as intracellular sensors of viral RNA [7,8,9]. The three predominant types of RLRs include: RIG-I, melanoma differentiation-associated gene 5 (MDA5), and laboratory of genetics and physiology 2 (LGP2). Both RIG-I and MDA5 function as primary sensors of viral RNA, with regulatory control overseen by LGP2 [6,7,8,9]. RIG-I can detect short double-stranded (ds) and single-stranded (ss) RNA sequences (i.e., 13–500 bp) that display either an unphosphorylated or phosphorylated 5′ region [7,8,9,10]. Unlike RIG-I, MDA5 is proficient at detecting long dsRNA sequences (i.e., >2000 bp) that exhibit an unphosphorylated 5′ region [8,10]. The formation of double-stranded RNA is a major part of the replication cycle of RNA viruses, so both RLRs are capable of sensing positive and negative sense RNA genomes [10]. Additionally, RLRs can detect RNA from some DNA viruses, such as the small RNA generated by the Epstein–Barr virus [8,9,11]. After binding with the viral RNA, RIG-I and MDA5 undergo conformational changes that expose their caspase activation and recruitment domain (CARD) [7,10]. CARD then interacts with mitochondrial antiviral signaling (MAVS) adapter proteins present in the mitochondria and initiates the tank-binding kinase 1 (TBK1)—IFN regulatory factor 3 (IRF3) signaling cascade [6,7,8,9,10]. After activation, MAVS binds to tumor necrosis factor receptor-associated factor 3 (TRAF3) and recruits three adaptor proteins: TRAF family member-associated nuclear factor-κ-B activator (TANK), NAK-associated protein 1 (NAP1), and similar to NAP1 TBK1 adaptor (SINTBAD) [6,7,12]. TRAF-associated adaptor proteins stimulate TBK1 and IκB kinase ε (IKKε) to phosphorylate the MAVS complex [6,7,12]. Afterward, the MAVS complex can attract IRF-3 or IRF-7 to become phosphorylated by TBK1 and IKKε [6,7,8,9,10,12,13]. Once phosphorylated, IRF-3/7 is translocated to the nucleus, where it can upregulate the expression of type-I IFNs [6,7,8,9,10,12]. Along with IRF-3/7, IκB also becomes phosphorylated, thereby activating nuclear factor-κB (NF-κB) [6,14]. After dissociating from IκB, NF-κB is translocated to the nucleus and upregulates the expression of proinflammatory cytokines, such as tumor necrosis factor α (TNFα), interleukin-2 (IL-2), IL-6, IL-8, and IL-12 [14,15].

### 1.4. Toll-like Receptor Signaling

Like RLRs, endosomal TLRs can detect viral nucleic acids and initiate the TBK1-IRF-3 and NF-κB signaling cascade [6,7,8,16]. Examples of endosomal TLRs include TLR3, TLR7, TLR8, and TLR9 [16]. Unlike RLRs, TLRs are predominantly expressed in cells of the immune system, such as dendritic cells, neutrophils, macrophages, B cells, and T cells [17]. Each endosomal TLR is specific to a different type of viral nucleic acid, with TLR3, TLR7/8, and TLR9 capable of sensing dsRNA, ssRNA, and dsDNA, respectively [16,17,18]. Viral nucleic acids become exposed to endosomal TLRs either by viral endocytosis or autophagy [16]. Activation of TLR3 follows a myeloid differentiation primary response 88 (MyD88) independent pathway, relying on the adaptor protein TIR-domain-containing adapter-inducing interferon-β (TRIF) [6,7,8,16,17,19]. After binding with viral dsRNA, TLR3 induces TRIF to recruit TRAF3, which then goes on to phosphorylate TBK1 and IKKε [6,7,8,16,17,19]. Both kinases then phosphorylate IRF-3/7 and IκB, thus upregulating the expression of type-I IFNs and proinflammatory cytokines [6,7,8,17,19]. Activation of TLR7/8 and TLR9 follows a MyD88-dependent pathway, which differs slightly from TLR3 [6,7,8,16,17,19]. After binding with the TLR, MyD88 is activated and forms a multiprotein complex with interleukin 1 receptor-associated kinase 4 (IRAK4), TRAF3, TRAF6, IRAK1, and IKKα [6,7,8,16,17,19]. In the protein complex, MyD88 recruits IRF7 to become phosphorylated by either IRAK1 or IKKα, while TRAF6 generates another complex with TGF-β-activated kinase 1 (TAK1) and TAK-binding protein 1/4 (TAB1/4) to phosphorylate IκB via the IKK complex [6,8,19]. The IKK complex consists of three proteins: IKKα, IKKβ, and NF-κB modulator (NEMO) [6,19].

### 1.5. IFN Signaling

**Figure 1:**
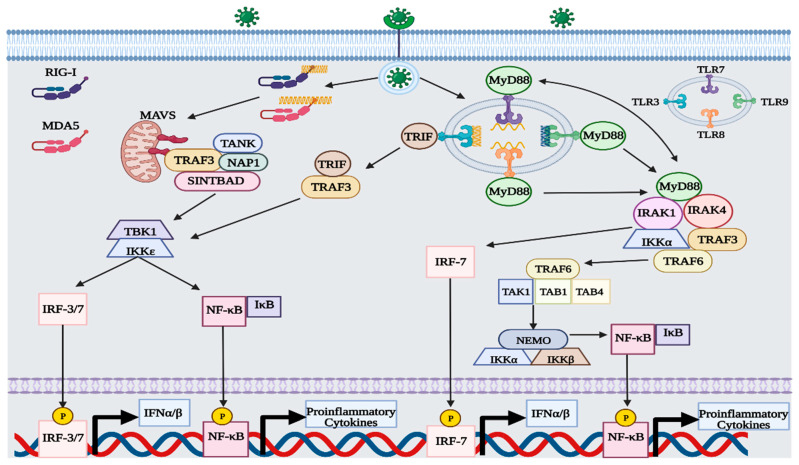
Overview of RLR and TLR pathway during viral infection. The recognition of viral nucleic acids by RLRs and TLRs induces activation of IRF-3/7 and NF-κB by phosphorylation, thus upregulating the expression of type-I IFNs and proinflammatory cytokines, respectively. This figure was created with BioRender.com (accessed on 20 January 2023).

**Figure 2:**
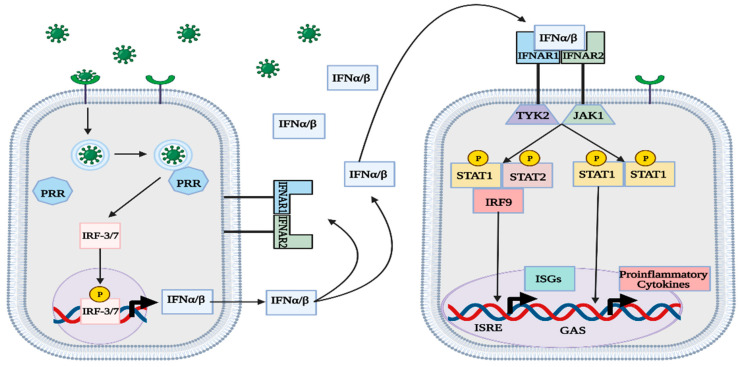
Overview of canonical type-I IFN pathway. PRRs within infected cells recognize the virus and induce IFNα/β production. The type-I IFNs are secreted and act on IFNα receptors, which facilitate the transcription of ISGs and proinflammatory cytokines. The generation of ISG proteins in uninfected cells confers greater protection against the virus. This figure was created with BioRender.com (accessed on 20 January 2023).

Although other mechanisms exist to activate the type-I IFN response, the signaling pathways employed by RLRs and TLRs are the most prevalent and thoroughly studied (Figure 1) [1,3,4,5,6,7,8,9,10,11,12,14,16,17,19,20,21]. IFNα/β is secreted out of infected cells to act on nearby IFNα receptors (IFNAR) in an autocrine and paracrine manner [1,3]. IFNAR consists of two subunits that are each associated with a different protein kinase: IFNAR1 with tyrosine kinase 2 (TYK2) and IFNAR2 with JAK1 [1,3,22]. After binding with IFNα/β, IFNAR induces phosphorylation of both TK2 and JAK1 [1,3,22]. In the canonical type-I IFN pathway, TYK2 and JAK1 activate STAT1 proteins by phosphorylation and promote the formation of different complexes that act as transcription factors for proinflammatory genes [1,3]. The IFN-stimulated gene factor 3 (ISGF3) complex, consisting of STAT1/2 and IRF-9, binds to an IFN-stimulated response element (ISRE) in the genome and facilitates the transcription of various IFN-stimulated genes (ISGs) [1,3,22]. Another notable complex is the phosphorylated STAT1 homodimer, also called IFN-γ activation factor (GAF), which binds to γ-activated sequences (GASs) and facilitates the transcription of proinflammatory genes (Figure 2) [1,3,22]. In the noncanonical type-I IFN pathway, TYK2 and JAK1 stimulate the transcription of ISGs without the use of STAT proteins, instead relying on mitogen-activated protein kinases (MAPKs) and phosphatidylinositol 3-kinase (PI3K) [1,3].

### 1.6. IFN-Stimulated Genes (ISGs)

The transcription of ISGs results in the production of numerous antiviral proteins that confer protection against viral infection [1,3,22,23]. ISG proteins guard against viruses by targeting different aspects of the virus replication cycle, namely the entry, translation, replication, or egress step [22,23,24,25]. Antiviral proteins that interfere with viral entry typically prevent the virus from releasing its genome into the cell’s nucleus or cytoplasm; however, they do not negate viral endocytosis [22,23]. Examples of anti-entry proteins include myxovirus resistance protein 2 (Mx2) and IFN-induced transmembrane protein 1-3 (IFITM1-3), which protect against human immunodeficiency virus 1 (HIV1) and influenza A H1N1 virus, respectively [26,27]. Other frequently studied ISGs include ISG15, IFN-induced tetratricopeptide repeat (IFIT) proteins, and viperin [22,23,28]. IFIT proteins are of particular note since their activity against viral translation has been shown to interfere with the replication of a diverse array of viruses.

## 2. IFN-Induced Tetratricopeptide Repeat (IFIT) Proteins

### 2.1. Expression of IFITs during Viral Infection

Characterized as an early product of the type-I IFN response, IFIT consists of a family of proteins that are expressed in the cytoplasm of many different species, from fish to mammals [29,30]. IFIT generally functions as an antiviral protein that can target viral RNA and inhibit the translation of viral proteins [22,23,29]. In placental mammals, cells can typically produce five types of IFIT: IFIT1, IFIT1B, IFIT2, IFIT3, and IFIT5 [29,30]. In humans, IFIT genes are located on chromosome 10, while in mice, IFIT genes are found clustered on chromosome 19 [30]. Although the location of IFIT genes differs between organisms, recent phylogenetic studies have shown that IFIT2, IFIT3, and IFIT5 are closely related between species [31]. Because of their similarities, mouse IFIT genes (Ifit) have been thoroughly studied to better understand the antiviral function of human IFIT genes [29,30].

### 2.2. Antiviral Functions of IFIT Proteins

Both human IFIT1 and IFIT2 (ISG56 and ISG54) and mouse Ifit1 and Ifit2 can inhibit the translation of viral proteins by binding to the eukaryotic initiation factor 3 (eIF3) complex, thus preventing the formation of the preinitiation complex [32,33,34]. IFIT1 and IFIT2 preferentially bind to the eIF3e subunit, while Ifit1 and Ifit2 act on the eIF3c subunit [32,33,34]. In humans, upregulated expression of IFIT2 has been seen during in vitro experiments with DNA and RNA viruses, such as adenovirus and influenza A virus [30]. Investigations of Ifit2 expression in mice have revealed that Ifit2 plays a crucial role in conferring protection against various neurotropic viruses, including vesicular stomatitis virus (VSV), West Nile virus (WNV), and mouse hepatitis virus strain A59 [30,35,36,37].

Unlike IFIT2, human IFIT3 (ISG60) functions as a major supporting protein that modulates the activity of other IFIT proteins [29,30,32,33,34]. IFIT3 upregulates the type-I IFN response by interacting with TBK1 and facilitating its activation by MAVS, thus increasing the expression of downstream ISGs [33,38]. Likewise, IFIT3 also binds to IFIT1, forming a heterotrimer consisting of IFIT1/2/3 to stabilize its structure and improve its binding against 2′O-unmethylated viral RNA [29,32,33,39]. During transcription, cellular RNA undergoes a three-step capping process: attachment of guanosine to the RNA’s 5′ region, N7-methylation of guanine (cap 0), and 2′O-methylation of the adjacent ribose (cap 1) [40]. In some mRNA, the two ribose adjacent to the guanine cap are 2′O-methylated (cap 2) [40]. Although most viruses exhibit cap 1 RNA to mimic host mRNA, some viruses can successfully infect their host with just cap 0 RNA, such as the hepatitis C virus (HCV) [40,41]. In contrast to mouse Ifit1, human IFIT1 alone cannot efficiently distinguish between host and viral RNA, so it exhibits the potential to weakly interact with phosphorylated, cap 0, and cap 1 RNA [29,30,31,32,33,34]. The unspecific binding affinity means that IFIT1’s capacity to inhibit viral replication mainly results from its interaction with eIF3, which reduces the translation of host and viral proteins [29,30,31,32,33,34,42,43]. The inability of IFIT1 to effectively target cap 0 RNA is resolved when it binds to IFIT3, which allows it to increase its efficacy against cap 0 RNA, while also retaining the ability to weakly recognize cap 1 RNA [39]. IFIT3 plays an important role in modulating the function of IFIT1; unfortunately, a similar function has not yet been shown in mouse Ifit3 [32,33,34,39]. Evidence of Ifit3 forming a heterotrimer with Ifit1/2 is still lacking; however, recent studies have shown that Ifit3 does exhibit antiviral activity, diminishing the replication of the rabies virus in C57BL/6 mice [44]. Mouse Ifit3b is another closely related IFIT3 protein sharing 96% sequence homology that has not been fully characterized [42].

**Figure 3:**
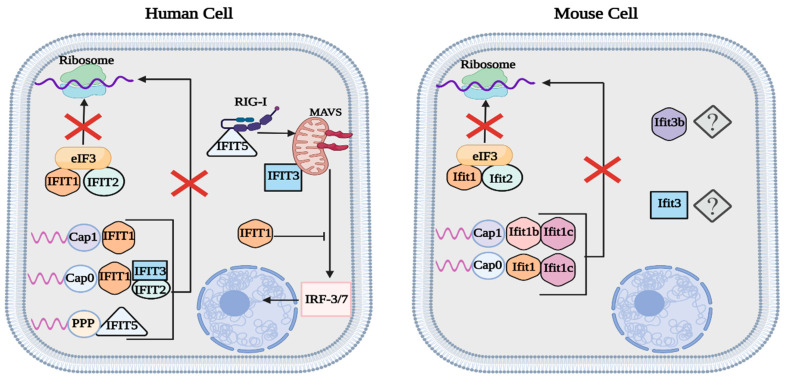
Summary of human and mouse IFIT function. Both human IFIT1/2 and mouse Ifit1/2 reduce viral protein translation by binding to eIF3, thus inhibiting the preinitiation complex. Similar to human IFIT3, mouse Ifit1c enhances the function of other IFIT proteins. IFIT1 is responsible for targeting cap 0/1 RNA in human cells, while mouse cells utilize Ifit1 and Ifit1b to target cap 0 and cap 1 RNA, respectively. IFIT5 binds to phosphorylated RNA and promotes the activity of MAVS. In contrast to IFIT3/5, IFIT1 acts as a negative regulator of the type-I IFN response. The function of mouse Ifit3 and Ifit3b has yet to be fully characterized. This figure was created with BioRender.com (accessed on 20 January 2023).

Important distinctions between human and mouse IFIT expression include the activity of IFIT1, IFIT1B, and IFIT5 [31,32,33]. Even though human IFIT1 and mouse Ifit1 have similar functions, Ifit1 is evolutionarily more closely related to IFIT1B because of its ability to distinguish between capped RNA [31]. Human IFIT1B has also been shown to target cap 0 RNA; however, the absence of an ISRE means that it is inactive during viral infection [42,43]. In mice, there are three main IFIT1B-related proteins: Ifit1, Ifit1b, and Ifit1c [31,32,33,34,42,43,45]. Ifit1 preferentially binds to cap 0 RNA, while Ifit1b selectively targets cap 1 RNA [31,45]. The activity of Ifit1 and Ifit1b is further enhanced after binding with Ifit1c [45]. The gene for IFIT5 is missing in mice but is present in humans and targets phosphorylated RNA that has not been capped [31,43,46]. Aside from its direct antiviral activity, human IFIT5 (ISG58) promotes the type-I IFN response by interacting with RIG-I and facilitating its binding to MAVS (Figure 3) [46]. The actions of IFIT3/5 are opposed to IFIT1, which functions as a negative regulator of the type-I IFN response [30,47].

## 3. IFIT Expression during Cancer

The expression of IFIT proteins is a hallmark of the type-I IFN response against viral infection; however, recent studies have elucidated a novel role for IFIT proteins in the context of nonviral pathologies, such as cancer [48]. Although cancer encompasses a diverse array of pathologies that are defined by cell type, metastasis is often characterized by an epithelial–mesenchymal transition (EMT) in neoplastic cells [49,50]. The resulting mesenchymal cells that are produced following EMT are less polarized and able to invade the basement membrane, thus allowing for tissue colonization via cardiovascular and lymphatic vessels [49,50]. Because EMT also occurs during embryogenesis and wound healing, the process can be initiated by various signaling pathways [49,50]. Of particular note, IFIT proteins have been shown to exhibit varying effects on the capacity of neoplastic cells to undergo EMT and metastasize [48].

The impact of IFIT protein expression on metastasis has been thoroughly explored in the context of oral squamous cell carcinoma (OSCC), which represents the sixth most prevalent cancer worldwide [48,51]. Studies examining human OSCC cell lines revealed that the presence of IFIT2 plays a major role in EMT and metastasis [48,52,53]. When IFIT2 is depleted from OSCC cells, they undergo EMT and metastasize at greater rates than control cells [52]. On the other hand, OSCC cells expressing IFIT2 in response to IFNβ treatment exhibited significantly less migration [53]. The in vitro findings resemble clinical data showing that patients with OSCC characterized by elevated IFIT2 expression experienced higher rates of postsurgical survival than OSCC patients with low IFIT2 expression, 66.5 months versus 18.9 months, respectively [53]. Evidence suggests that IFIT2 reduces the migration potential in OSCC by interfering with atypical protein kinase C (aPKC) signaling [52]. While the absence of IFIT2 was shown to promote the activation of aPKC and induce EMT during OSCC, IFIT2’s function as a proapoptotic factor may also result in increased cancer survival when present in neoplastic cells [52,53,54,55]. IFIT2 expression induces activation of caspase 3 and promotes apoptosis via the intrinsic pathway [54]. Increased apoptosis from IFIT2 stimulation has been accomplished in cancer cell lines treated with proteasome inhibitors [55]. When the proteasome is inhibited, IFIT2 is allowed to aggregate in the centrosome and induce apoptosis [55]. While the function of IFIT2 in other cancers is still under investigation, current findings indicate that IFIT2 plays a negative role in neoplastic cells by reducing migration and promoting apoptosis [48,52,53,54,55].

Highlighting the diversity between IFIT proteins, IFIT1 and IFIT3 are distinguished from IFIT2 for their pro-metastatic action in the setting of OSCC [48,56]. Recent studies examining IFIT1 and IFIT3 expression in OSCC cell lines revealed that it was associated with EMT and increased cell invasion [48,56]. Overexpression of IFIT1/3 results in unregulated recycling of phosphorylated epidermal growth factor (p-EGFR), which promotes increased expression of EMT-related markers, such as N-cadherin [56]. While the overexpression of IFIT1 and IFIT3 seems to contribute to metastatic disease, its effect on p-EGFR results in increased susceptibility to chemotherapeutic medications that inhibit tyrosine kinases, such as gefitinib [56]. OSCC cell lines pretreated with IFNα prior to gefitinib treatment showed elevated IFIT1 and IFIT3 expression and increased drug susceptibility [56]. Along with their role in OSCC, IFIT1 and IFIT3 have recently been shown to contribute to the progression of pancreatic cancer, which exhibits a 5-year overall survival rate of about 7% [48,57,58,59,60]. Analysis of IFIT1 expression in pancreatic cancer cell samples showed that that it was overexpressed in relation to normal pancreatic cell samples [57]. When IFIT1 was tested in pancreatic cancer cell lines, the cells overexpressing IFIT1 exhibited a greater capacity for invasion than IFIT1 knockout cells [57]. The capacity of IFIT1 to promote EMT in pancreatic cancer cell lines is thought to be caused by the Wnt/β-catenin pathway, which is upregulated during IFIT1 overexpression [57]. Similar to IFIT1, IFIT3 also contributes to the progression of pancreatic cancer [58,59,60]. A retrospective study analyzing patient pancreatic cancer cells revealed that patients undergoing neoadjuvant therapy exhibited longer disease-free survival times if they had IFIT3-negative cancer cells [59]. The role of IFIT3 in pancreatic cancer was corroborated in later in vitro studies, which showed that IFIT3 promotes metastasis [58]. Unlike IFIT1, IFIT3 promotes metastasis in pancreatic cancer cells by inhibiting the proapoptotic effects of IFIT2 and by upregulating the secretion of vascular endothelial growth factor (VEGF) and IL-6 [58]. Increased levels of VEGF improve vascularity in the tumor tissue, while IL-6 promotes an inflammatory environment that is known to facilitate tumor growth [58]. IFIT3’s effect on chemotherapy treatment in pancreatic cancer is opposite to its effect in OSCC [60]. In the setting of pancreatic cancer, IFIT3 expression increases resistance against chemotherapy agents, such as gemcitabine and paclitaxel [60]. Resistance to these medications results from IFIT3’s binding to the mitochondrial protein voltage-dependent anion-selective channel protein 2 (VDAC2), which inhibits treatment-induced apoptosis [60]. Differences in IFIT3’s effect on chemotherapy susceptibility may be due to the drug’s mechanism of action, with gemcitabine and paclitaxel relying on compounds that disrupt DNA replication and mitosis, respectively.

Although not as well characterized as IFIT1/2/3, IFIT5 has also been implicated in the progression of cancer, specifically bladder and prostate cancer [48,61,62]. When comparing IFIT5 expression levels between nonmuscle-invasive and muscle-invasive bladder cancer samples, the invasive cells exhibited significantly higher levels of IFIT5 expression [61]. Further in vitro testing using bladder cancer cell lines corroborated the analysis of patient samples, with cells overexpressing IFIT5 exhibiting EMT and increased migration [61]. IFIT5-induced EMT is thought to be due to its inhibitory effect on miR-99a, a microRNA that negatively regulates EMT-associated transcription factors [61]. A similar effect is seen in prostate cancer, where IFIT5 expression induced by IFNγ treatment results in the inhibition of miR-363, which is another negative regulator of EMT [62].

## 4. IFIT Expression during Sepsis

As seen in the context of cancer, the roles of IFIT proteins have expanded past the classical view of being purely antiviral agents and are now understood to participate in different disease states. One area of research that is currently gaining steam is the study of IFIT proteins in the context of sepsis. Contemporary definitions of sepsis characterize it as a deleterious host response toward a pathogen that can result in lethal organ dysfunction [63,64]. A major inciting factor during sepsis is the initiation of an exaggerated immune response, leading to the dysregulated release of proinflammatory cytokines [63]. The heightened inflammatory state negatively affects hemostatic stability, promoting systemic endothelial damage that causes widespread activation of clotting factors [63,65]. Systematic activation of the clotting pathway can lead to disseminated intravascular coagulation (DIC), resulting in thrombi formation that blocks blood flow, thus increasing the risk of multiple organ failure (MOF) [63,65]. Along with endothelial damage, the inflammatory response can also promote vascular instability by facilitating vascular leakage of plasma into the surrounding interstitium [63,65]. When the ensuing drop in blood pressure becomes resistant to therapeutic interventions, then the diagnosis of sepsis is altered to septic shock [63,65]. Even with prompt medical treatment, the mortality rate of sepsis and septic shock is still exceedingly high, with current estimations at 15–20% and 20–50%, respectively [63].

Because sepsis is initiated by an invading pathogen, it can be caused by bacteria, fungi, or viruses [66,67]. Although the secretion of IFNα/β is most closely associated with a viral infection, some PRRs that recognize bacteria and fungi, such as TLR4, can also induce IFNα/β secretion via a TRIF-dependent pathway [29,68,69]. Bacteria-induced sepsis, or bacterial sepsis, represents over 60% of all diagnosed cases of sepsis [66,67]. Due to its oversized representation in sepsis, bacterial sepsis has been thoroughly studied in both in vitro and in vivo models. The role of IFIT2 during bacterial sepsis is still under investigation, with recent studies providing conflicting results [29,70,71]. In vitro studies examining IFIT2 overexpression in mouse macrophages that were previously stimulated with LPS revealed that increased IFIT2 was associated with significantly reduced secretion of TNFα and IL-6 [29,70]. When tested in a mouse model, the absence of IFIT2 was associated with less secretion of TNFα and IL-6 and significantly improved survival outcomes against LPS-induced septic shock [29,71]. The different effects of IFIT2 in both studies may be due to the complex nature of sepsis, which may not be accurately represented by single-cell line experimentation. Fungal sepsis constitutes the second most common form of sepsis, characterizing about 20% of all cases [66,67]. Similar to bacterial sepsis, IFIT2 knockout mice exhibited increased survival when challenged against a systemic *Candida albicans* infection [29,72].

Although not considered a common cause of sepsis, the study of viral sepsis has gained a renewed interest in response to the coronavirus disease 2019 (COVID-19) pandemic. Before the recent coronavirus pandemic, common causes of viral sepsis only included enteroviruses, human parechoviruses, herpes simplex virus, dengue virus, adenovirus, and influenza virus [63,73]. Severe acute respiratory syndrome coronavirus 2 (SARS-CoV-2), the causative agent of COVID-19, has emerged as a novel cause of viral sepsis, with 76% of septic COVID-19 patients reporting negative cultures for bacteria or fungi [63,74]. As expected with other viral infections, transcriptomic analysis of COVID-19 patient samples revealed that the type-I IFN response was upregulated, with significant increases in IFIT protein mRNA [75]. To counteract the antiviral effect of host IFIT proteins, SARS-CoV-2 is equipped with viral proteins that can reduce the type-I IFN response via different mechanisms [76,77]. For instance, open reading frame 3b (ORF3b) can inhibit the initiation of the type-I IFN response by preventing the translocation of IRF3, thus reducing the production of IFNα/β [76,77]. The type-I IFN response is further inhibited by nonstructural protein 1 (Nsp1), which interacts with ribosomes and prevents the translation of host mRNA, thereby cutting down the production of IFIT protein and other antiviral factors [77]. The importance of IFIT proteins in clearing coronavirus infections is evidenced by the immune evasion strategies employed by SARS-CoV-2 and previous animal studies [37]. When challenged against the coronavirus mouse hepatitis virus A59, IFIT2 knockout mice exhibited increased viral loads and higher mortality rates when compared with wild-type mice [37]. Further research is required to examine the role of other IFIT proteins during the course of viral sepsis.

## 5. Conclusions

The type-I IFN response is instrumental to the innate immune defense against viral pathogens. While the type-I IFN response is associated with the production of numerous proinflammatory cytokines and antiviral agents, the role of IFIT proteins has been extensively characterized in both humans and mice. Recent studies examining IFIT proteins have revealed that they affect the progression of nonviral pathologies, such as cancer and sepsis. Although more work is required to accurately elucidate the impact of IFIT proteins in cancer, recent investigations reveal that IFIT1, IFIT3, and IFIT5 promote metastasis, while IFIT2 acts against neoplastic cells by promoting apoptosis. The role of IFIT proteins in bacterial/fungal sepsis is controversial; however, its effect during viral sepsis is more defined. The capacity of viral pathogens to become systemic and cause viral sepsis is facilitated by using viral proteins that inhibit the type-I IFN response. SARS-CoV-2, in particular, has emerged as a novel cause of viral sepsis due to its wide arsenal of viral proteins that diminish the translation of IFIT proteins. By understanding the role of IFIT proteins in different pathologies, new therapeutics can be developed to target their action. For instance, during OSCC, medications that downregulate the production of IFIT proteins may help reduce mortality rates by decreasing the rate of metastasis. In the case of viral sepsis, medications that upregulate the production of IFIT proteins may facilitate viral clearance and prevent progression to septic shock. Going forward, studies examining IFIT proteins should continue to explore their effect on different pathologies and investigate medications that have the potential to affect their production.

## Data Availability

Not applicable.
